# Molecular features of steroid-binding antidins and their use for assaying serum progesterone

**DOI:** 10.1371/journal.pone.0212339

**Published:** 2019-02-20

**Authors:** Nitin Agrawal, Soili I. Lehtonen, Meri Uusi-Mäkelä, Purvi Jain, Sari Viitala, Juha A. E. Määttä, Niklas Kähkönen, Latifeh Azizi, Tiina A. Riihimäki, Markku S. Kulomaa, Mark S. Johnson, Vesa P. Hytönen, Tomi T. Airenne

**Affiliations:** 1 Structural Bioinformatics Laboratory, Biochemistry, Faculty of Science and Engineering, Åbo Akademi University, Turku, Finland; 2 BioMediTech Institute and Faculty of Medicine and Health Technology, Tampere University, Tampere, Finland; 3 University of Eastern Finland, School of Pharmacy, Kuopio, Finland; 4 Fimlab Laboratories, Tampere, Finland; Okayama Daigaku, JAPAN

## Abstract

Chicken avidin (Avd) and streptavidin from *Streptomyces avidinii* are extensively used in bionanotechnology due to their extremely tight binding to biotin (K_d_ ~ 10^−15^ M for chicken Avd). We previously reported engineered Avds known as antidins, which have micro- to nanomolar affinities for steroids, non-natural ligands of Avd. Here, we report the 2.8 Å X-ray structure of the sbAvd-2 (I117Y) antidin co-crystallized with progesterone. We describe the creation of new synthetic phage display libraries and report the experimental as well as computational binding analysis of progesterone-binding antidins. We introduce a next-generation antidin with 5 nM binding affinity for progesterone, and demonstrate the use of antidins for measuring progesterone in serum samples. Our data give insights on how to engineer and alter the binding preferences of Avds and to develop better molecular tools for modern bionanotechnological applications.

## Introduction

Due to the highly stable beta-barrel structure, high resistance to a wide range of temperature and pH changes, rather small size (around 60 kDa for a tetramer) and, especially, the extraordinary tight binding to a small molecule D-biotin, avidins (Avds) are exploited in a number of practical life science applications and biotechnological assays. These include techniques for imaging, purification, labeling, targeting and detection [1,2]. The structure-function relationship of the Avd-biotin complex is well known, especially for the classical tetrameric chicken Avd and streptavidin from *Streptomyces avidinii*, and has been widely explored *e*.*g*. through rational mutagenesis [3]. Several Avds with altered biological and physiochemical properties have been developed and produced [1,3–5], including *e*.*g*. Avds with higher thermal stability [4,6]; single-chain and dual-chain Avds–circularly permuted Avds consisting of four or two monomers, respectively, fused into a single polypeptide chain–that have potential to bind simultaneously up to four different ligands as well as monomeric avidins, enabling applications where oligomeric assembly is disadvantageous [5,7–11]. Despite intensive research focusing on Avd structure and function, the biological role of Avd remains partially unclear. In chicken, Avd expression is induced by injury and inflammation [12] and Avd appears to be beneficial for the developing embryo in birds [13]. Numerous microbial genomes also carry an Avd gene, which may offer a tool for bacteria *e*.*g*. to compete against organisms incapable of biotin synthesis, such as nematodes in soil [14], and we can speculate on potential metabolic advantages to plants like soy bean having a symbiotic relationship with *Bradyrhizobium diazoefficiens*–a bacteria having the gene.

Currently, the number of stable and high-affinity small molecule-binding protein-based scaffolds for biotechnological use is still limited [15–17]. While several scaffolds appear suitable for binding of peptide/protein ligands, *e*.*g*. anticalins tailored from lipocalins of the calycin protein superfamily to bind small hapten-like compounds and large protein antigens [18], scaffolds suitable for tight binding of small molecules are rare. The Avd scaffold provides an optimal–in terms of both stability and physicochemical properties–ligand-binding site for small molecules; the binding site is lined with several polar residues available for hydrogen-bonding (H-bonding) and aromatic and hydrophobic residues for providing stability through van der Waals contacts and hydrophobic interactions [19]. Since the discovery of the extremely tight Avd-biotin interaction [20,21], the search for ligands other than biotin that could also bind Avds has been going on and has resulted in the identification of ligands, such as HABA (2-(4’-hydroxybenzene)azobenzoic acid) and its derivatives–a group of azo compounds binding to Avd [22] and streptavidin [23] with a micromolar affinity (K_d_ ≈ 6 × 10^−6^ M for Avd-HABA complex). In addition, several bacterial Avds, such as streptavidin [24], bradavidin [25] and hoefavidin [26], have intrinsic peptide ligands formed from their extended C-terminal regions that bind to their ligand-binding sites in the absence of biotin, typically only with moderate (micromolar) affinity. Peptide ligands have also been engineered and include tags like Avi- and AviD-tag [27,28], Strep-tag [29], Strep-tag II [29–33], different Nano-tags [34,35] and SBP-tag [36] with affinities on the micromolar (Avi-, AviD-, Strep-tag I and II) to nanomolar (Nano-tags) scale.

The binding site of Avds has been engineered by both mutating single residues involved directly in biotin binding and by manipulating the loop regions of Avds important for ligand recognition and specificity [37,38]. The L3,4-loop in particular, with a vital role for biotin binding, has been an active target for mutagenesis [5,7–10]. In our recent study, we reported antidins, *i*.*e*. engineered Avds recognizing the non-native ligands progesterone, testosterone, hydrocortisone, cholic acid and ketoprofen. Those antidins were created by engineering the L1,2-loop together with the L3,4-loop or L5,6-loop regions using a directed evolution method for mutagenesis and the phage display technique for selection [38]. In comparison to natural Avd, antidins showed reduced binding affinity for biotin and had increased affinity towards progesterone, as analyzed *in vitro* using calorimetry, fluorometry and microplate assays, and hence antidins have potential to be used as novel diagnostic reagents [38].

Here, we co-crystallized the sbAvd-2 (I117Y) antidin together with progesterone [PDB:5LUR]–the sbAvd-2 (I117Y) mutant has modified L1,2 and L3,4 loop regions and includes the stabilizing mutation I117Y [22] not present in chicken Avd. We created new synthetic phage display libraries and report the fluorometry-based progesterone-binding analysis of new enriched antidins, and of recently reported ones. We focus in more detail on one of our most potent new antidins, sbAvd-7, and demonstrate its potential use for assaying the concentration of progesterone in serum samples. Moreover, homology modelling and docking analysis was conducted to enhance our understanding of the experimental observations at atomic resolution. Our data help us to better understand the binding mode of progesterone with antidins and may help in developing improved forms of antidins for biotechnological applications, including for diagnostics.

## Results

### Overall structure of sbAvd-2 (I117Y)—Progesterone complex

**Fig 1:**
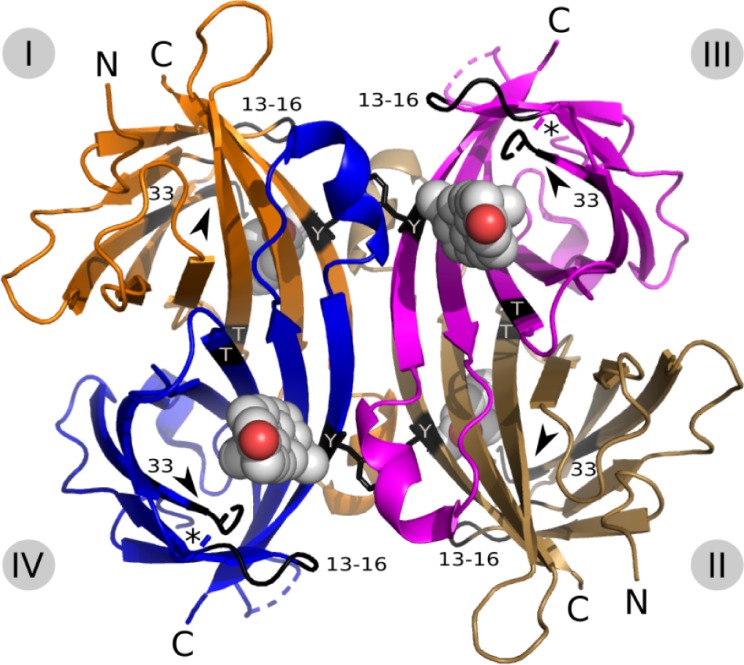
Overall structure of the sbAvd-2 (I117Y)-progesterone complex [PDB:5LUR]. Subunits I (orange), II (brown), III (magenta) and IV (blue) of the biological unit are numbered according to [19]. The position of residues Arg13-Met14-Asn15-His16 (13–16); Tyr33 (33); Ala35-Thr36-Val37-Asn38 (arrow head); Thr77 (T); and Tyr117 (Y; stick model) are indicated with black color in the cartoon models of the subunits I-IV. The progesterone ligands are drawn as spheres; only the oxygen atoms (red) of the 20-acetyl group (D-ring) of subunits III and IV are clearly seen. The N-termini (N; * for subunits II and III) and C-termini (C) are indicated. The positions of the residues in the L3,4 and L6,7 loop that could not built into the final structure due to uninterpretable electron density map are indicated by dashed lines.

**Fig 2:**
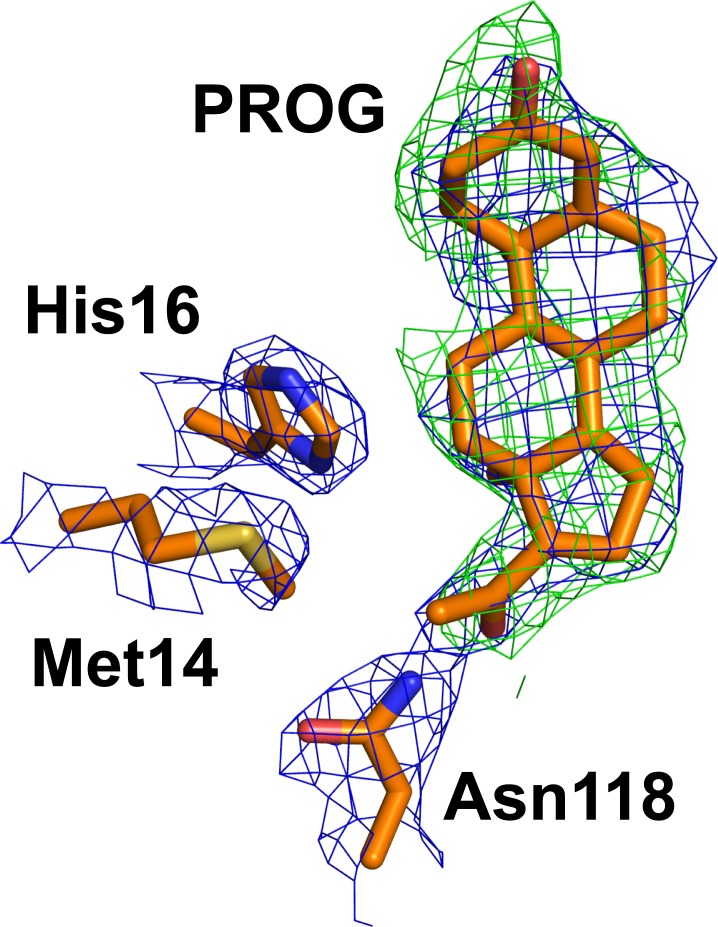
Progesterone interaction with the key residues Met14, His16 and Asn118 of the sbAvd-2 (I117Y)-progesterone complex [PDB:5LUR]. The weighted 2Fo-Fc electron density map (blue mesh; contour level of 1.0σ) and an omit map (green mesh; contour level of 1.0σ) calculated in the absence of progesterone are shown. Carbon atoms are colored orange, nitrogen atoms blue, sulfur atom yellow and oxygen atoms red.

**Fig 3:**
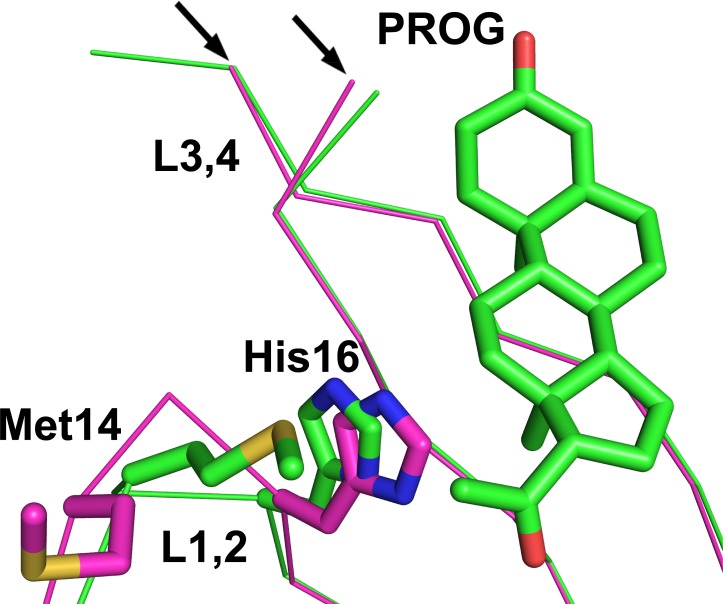
Superimposition of the Cα traces of subunit I of the sbAvd-2 (I117Y)-progesterone complex (green carbon atoms) [PDB:5LUR] and the apo structure of sbAvd-2 (I117Y) (magenta carbon atoms) [PDB:4U46]. Progesterone [PDB:5LUR] and the side chains of Met14 and His16 [PDB:5LUR, 4U46] are shown as sticks. The L1,2 and L3,4 loop regions are labeled. Black arrows indicate the L3,4 loop regions missing (uninterpretable electron density) from both of the structures. Nitrogen atoms are colored blue, oxygen atoms red and sulfur atoms yellow.

The sbAvd-2 antidin was previously shown to bind progesterone with 111 nM affinity. The I117Y mutation, originating from avidin-related protein 4 (AVR4) [39] and known to stabilize the 1,3 interface of tetrameric Avds, slightly decreased the affinity (180 nM) for progesterone while improving the thermal stability. Here, the X-ray structure of sbAvd-2 (I117Y) in a putative complex with progesterone [PDB:5LUR] was determined at 2.80 Å resolution (see Table 1 for structure determination statistics; Fig 1). This antidin was co-crystallized with progesterone and the two binding sites of the asymmetric unit have clear blobs of electron density, most likely representing progesterone molecules even though the density at the binding sites was worse, or less interpretable, than for most regions of the polypeptide chains (Fig 2). The biological unit was clearly tetrameric despite there being only two monomers in the asymmetric unit; subunits I-IV were numbered according to [19]. Similarly to our recently published apo structure of sbAvd-2 (I117Y) [PDB:4U46], the overall fold of the complex structure closely resembled that of chicken Avd [19]. Residues Asn38-Ser41 within the L3,4 loop were missing from the final structure; they could not be built because of the lack of interpretable electron density for these residues (Fig 3).

**Table 1:** Structure determination statistics for [PDB:5LUR].

DATA PROCESSING^a^	
Space group	P2_1_2_1_2
Unit cell:	
a, b, c, (Å)	74.48, 79.80, 43.07
α, β, γ (°)	90, 90, 90
Wavelength (Å)	0.967700
Beamline	ID30A-3, ESRF
Resolution (Å)^b^	25–2.80 (3.0–2.80)
Observed Reflections^b^	28590 (4922)
Unique Reflections^b^	7466(1249)
I/sigma^b^	10.36 (3.22)
R_factor_ (%)^b^	12.0 (41.7)
Completeness^b^	96.1 (98.5)
REFINEMENT	
Matthews coefficient	2.10
R_work_ (%)^c^	17.6%
R_free_ (%)^c^	23.9%
Monomers (asymmetric unit)	2
R.m.s.d:	
Bond lengths (Å)	0.0125
Bond angles (°)	1.71

^a^The numbers in parenthesis refer to the highest resolution bin

^b^Data from XDS [40]

^c^Data from Refmac 5 [41]

#### Progesterone-binding mode of sbAvd-2 (I117Y) mutant

**Fig 4:**
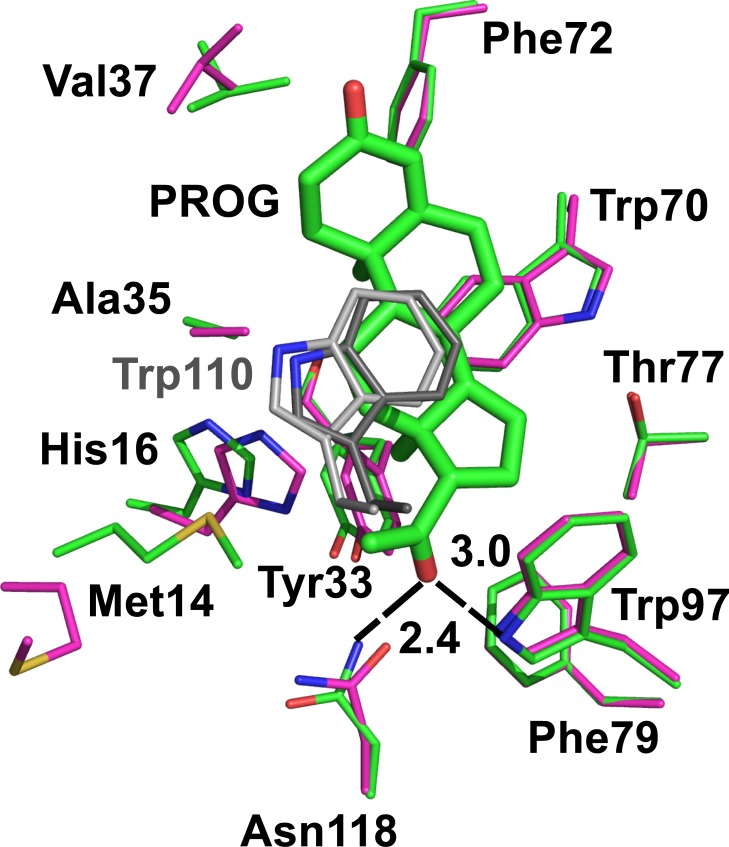
Comparison of the ligand-binding site of the sbAvd-2 (I117Y)-progesterone complex structure (green; subunit I) [PDB:5LUR] and the apo structure of sbAvd-2 (I117Y) (magenta) [PDB:4U46]. Residues within 4 Å around the ligand (thick sticks) in the sbAvd-2 (I117Y)-progesterone complex structure, and the equivalent residues in the apo structure are shown as sticks; the Cα traces of the subunits were superimposed. The side chain carbon atoms of Trp110 from the neighboring subunit of the complex structure are colored dark grey and, of the apo structure, light grey. Nitrogen atoms are colored blue, oxygen atoms red and sulfur atoms yellow. The H-bonds between progesterone and side chains of Trp97 and Asn118 of the complex structure are indicated by black dashed lines (distances in Ångströms).

Unlike the biotin ligand in the chicken Avd–biotin complex [PDB:2AVI], which is stabilized by eleven hydrogen bonds and several hydrophobic/van der Waals interactions [19], in our new sbAvd-2 (I117Y)–progesterone complex structure the ligand has only a few residues within 4 Å to stabilize its binding (Fig 4). Firstly, only two H-bonds are formed between sbAvd-2 (I117Y) and progesterone: the Asn118 Nδ (2.4 Å) and Trp97 Nε (3.0 Å) atoms form H-bonds with the 20-acetyl group (D-ring) of the ligand; by comparison, in chicken Avd [PDB: 2AVI] only Asn118 Oδ (2.8 Å) is H-bonded to the ureido ring of biotin while Trp97 Nε (3.9 Å) remains unbound. The oxygen atom of the ketone group at the other end of the progesterone (A-ring) is not forming H-bonds with the surrounding residues. Secondly, the Cδ and Cζ atoms of Phe79 are within a van der Waals and/or hydrophobic interaction distance (3.7 Å and 3.5 Å, respectively) of the 20-acetyl group of the ligand; and Met14, His16, Tyr33, Ala35, Val37, Trp70, Phe72, Thr77 and Trp110 (from the neighboring subunit) stabilize the binding of progesterone through van der Waals and hydrophobic interactions. All-in-all, the fit of progesterone to sbAvd-2 (I117Y) in the complex is much less optimal than biotin in the Avd-biotin structure; and progesterone does not stabilize the L3,4-loop (see below), in agreement with the lower binding free energy of steroids to sbAvds in comparison to the binding of biotin to Avd.

#### L1,2 and L3,4 loop

Dissimilar to the apo structure of sbAvd-2 (I117Y) [PDB:4U46] published previously [38], the L1,2 loop of the sbAvd-2 (I117Y)–progesterone structure is clearly stabilized in one major conformation, very likely needed to accommodate ligand binding: in the antidin-progesterone complex, Met14 and His16 of the L1,2 loop have clearly moved (in comparison to the apo structure) towards the ligand and are within hydrophobic/van der Waals interaction distance of the ligand (Fig 3). However, the L3,4 loop of the progesterone complex structure is in the open conformation similar to the apo structure of sbAvd-2 (I117Y) [PDB:4U46]. This suggest that further sequence optimization of the L3,4 loop could result in the closed conformation of the loop and improve the affinity between progesterone and the antidin.

#### The I117Y mutation

We reported recently [38] that the I117Y point mutation improves the thermostability of sbAvd antidins similarly to what was originally shown for chicken Avd [22]. This form was found to crystallize in the presence of progesterone and was thus selected for structural analysis. The I117Y mutation slightly reduced the affinity of progesterone to sbAvd-2 and it is possible that the Tyr117 residue in the mutant, located at the 1,3 subunit interface, affects the binding of progesterone indirectly by altering the conformation/spatial location of the adjacent Asn118.

### Improved phagemid vector for phage display

To search for better binding antidins, we used phage display with an improved phagemid vector. The previously constructed and used Gateway cloning compatible phagemid vector, pGWphagemid [42], is based on the *ccdB* suicide gene that is not optimal when combined with F-episome bearing bacteria essential for phage display; F-episome bearing bacteria such as XL1-Blue are capable of producing the ccdA antidote, which prevents selection with *ccdB* to some extent [43], potentially leading to non-expressing bacterial clones and decreased screening efficacy. Therefore, we replaced the suicide gene *ccdB* with the *SacBR* gene, which has previously been used in plant cells immune to the ccdB toxin [44]. The resulting pGWSacBRphagemid vector was then successfully used for efficient screening of novel antidins using phage display (S1 Fig).

### Novel Avd phage display libraries

Two novel DNA libraries, AvLib-4 and AvLib-5, were created to screen for novel binders for progesterone and hydrocortisone, respectively, based on the previously enriched antidin sequences [38]. The constructed Avd phage display libraries were based on the monovalent display mode (3+3) and, due to the amber stop codon placed in between the Avd variants and the C-terminal pIII, the Avd variants could be expressed as tetramers on the surface of phages as described in [42]. The use of TRIM (trinucleotide mutagenesis) technology within the chemical synthesis process enabled preparation of DNA libraries with maximal diversity and good sequence coverage with a reasonable library size. After three rounds of selection with an automated magnetic particle processor, four antidins–sbAvd-7–10 –having mutations in the L1,2-loop and L3,4-loop regions (and at position 77; in comparison to chicken Avd) were selected from the progesterone panning AvLib-4 library, and six antidins–hbAvd-4–9 –having mutations in the L1,2-loop and L5,6-loop (and at positions 33, 40 and 118) were selected from the hydrocortisone panning AvLib-5 library (Table 2). Panning for hydrocortisone was also carried out using a 1:1 mixture of the AvLib-4 and AvLib-5 libraries but the hydrocortisone binders were dominantly enriched from the AvLib-5 library.

**Table 2:** Characteristics of AvLib library design and enriched sequences selected from the libraries based on ligand panning.

	L1,2 loop					L3,4 loop						L5,6 loop								
**Library**	**N12**	**D13**	**L14**	**G15**	**S16**	**Y33**	**T35**	**A36**	**V37**	**T38**	**T40**	**F72**	**S73**	**S75**	**T77**	**I117Y**	**N118**	**Template gene**	**Library size (theoretical / actual**^a^**)**	**INPUT**
AvLib-3 [34]	-	R	M	N	H	-	NNY degenerate codon				-	-	-	-	-	-	-	sbAvd-1	0.05 x 10^6^/	
							enables 15/20 amino												1.40 x 10^6^	
							acids (excluding W,													
							Q, E, M, K)													
**AvLib-4**^b^	-	R	M	N	H	all	T	A	V	Y	-	-	-	-	all	-	all	sbAvd-1	2.74 x 10^6^/	
						except	Y	T	D	N					except		except		1.11 x 10^9^	
						C	P	Y	L	P					C		C			
							A	S	S	S										
								P	H											
**AvLib-5**^c^	N	D			S	all					all	A	T	A			all		0.66 x 10^6^/	
	Y	S	-	-	A	except	-	-	-	-	except	Y	A	Y	-	-	except	wtAvd	3.33 x 10^6^	
						C					C		Y				C			
**Mutant**	**N12**	**D13**	**L14**	**G15**	**S16**	**Y33**	**T35**	**A36**	**V37**	**T38**	**T40**	**F72**	**S73**	**S75**	**T77**	**-**	**N118**	**From library**	**Target molecule**	**OUTPUT**
**hbAvd-4**	-	-	-	-	A	F	-	-	-	-	P	A	Y	Y	-	-	A	AvLib-5	hydrocortisone	
**hbAvd-5**	-	S	-	-	A	H	-	-	-	-	D	A	Y	A	-	-	S	AvLib-5	hydrocortisone	
**hbAvd-6**	-	-	-	-	A	L	-	-	-	-	F	Y	Y	Y	-	-	G	AvLib-5	hydrocortisone	
**hbAvd-7**	-	-	-	-	A	I	-	-	-	-	W	A	Y	Y	-	-	A	AvLib-5	hydrocortisone	
**hbAvd-8**	-	-	-	-	A	-	-	-	-	-	N	A	Y	Y	-	-	L	AvLib-5	hydrocortisone	
**hbAvd-9**	-	S	-	-	A	A	-	-	-	-	K	Y	A	Y	-	-	A	AvLib-5	hydrocortisone	
**sbAvd-7**	-	R	M	N	H	-	A	S	L	N	-	-	-	-	A	-	-	AvLib-4	progesterone	
**sbAvd-8**	-	R	M	N	H	F	-	Y	S	Y	-	-	-	-	A	-	-	AvLib-4	progesterone	
**sbAvd-9**	-	R	M	N	H	-	Y	Y	L	P	-	-	-	-	A	-	-	AvLib-4	progesterone	
**sbAvd-10**	-	R	M	N	H	F	Y	A	D	P	-	-	-	-	G	-	-	AvLib-4	progesterone	
sbAvd-1 [38]	-	R	M	N	H	-	-	-	-	-	-	-	-	-	-	-	-	AvLib-3	progesterone	Previously
sbAvd-2 [38]	-	R	M	N	H	-	A	T	V	N	-	-	-	-	-	-	-	AvLib-3	progesterone	Characteri-
sbAvd-2(I117Y) [38]	-	R	M	N	H	-	A	T	V	N	-	-	-	-	-	Y	-			zed antidins
sbAvd-3 [38]	-	R	M	N	H	-	P	A	D	P	-	-	-	-	-	-	-	AvLib-3	progesterone	
sbAvd-4 [38]	-	R	M	N	H	-	P	Y	L	S	-	-	-	-	-	-	-	AvLib-3	progesterone	
sbAvd-5 [38]	-	R	M	N	H	-	V	P	H	P	-	-	-	-	-	-	-	AvLib-3	progesterone	
sbAvd-6 [38]	-	R	M	N	H	-	V	S	S	N	-	-	-	-	-	-	-	AvLib-3	progesterone	

^a^Calculated from transformation activity

^b^Library design based on the enriched sequences with affinity towards progesterone and testosterone [38], sbAvds

^c^ Library design based on the enriched sequences with affinity towards hydrocortisone and cholic acid [38], hbAvds and cabAvds

In comparison to our previous study [38], progesterone-binding antidins were here selected from AvLib-4 library by using free progesterone for the elution in the first panning round followed by elution by acid in the subsequent rounds. The idea was to favor the selection of target-specific binders over non-specific binders during the initial selection from the library, after which the binders should already show desired target specificity [45]. Non-specific elution was used on the 2^nd^ and 3^rd^ panning rounds, followed by small-scale amplification of the phages [38]. The enriched antidins showed significantly improved affinities for progesterone and, for the first time, an antidin (sbAvd-7) with K_d_ < 10 nM for progesterone was enriched.

Different phage elution strategies (acid, base, competing ligand and trypsin elution) were tested for hydrocortisone selection to achieve high-affinity clones–the most unique sequences were obtained by using free hydrocortisone. However, none of the new binders showed a significantly higher affinity for hydrocortisone in comparison to the binders reported earlier by [38] (the affinities were in the micromolar range) but instead they showed improved affinity for progesterone (see below). It might be that the conjugated hydrocortisone molecule used in the selection process was either too large to fit properly into the ligand-binding site of the mutated Avds or, alternatively, the conjugation site in hydrocortisone was not optimal and affected ligand binding. Nonetheless, these results demonstrate that it is possible to select progesterone binders not only from Avd phage display libraries specifically having the “RMNH” sequence replacement in the L1,2-loop but also from other libraries. Since the affinity for hydrocortisone was not improved in the antidins enriched from the AvLib-5 in comparison to our earlier study [38], we will focus here on the analysis of novel progesterone-binding antidins.

### Progesterone-binding analysis of selected antidins

In our earlier study [38], the sbAvd-1–6 antidins, as well as the sbAvd-2 (I117Y) mutant, were experimentally tested for biotin, progesterone and testosterone binding using non-conjugated ligands in a fluorometry-based assay [38]; sbAvd-2 (K_d_ = 111 nM), sbAvd-5 (108 nM) and sbAvd-6 (117 nM) were observed as the tightest progesterone binders. These sbAvds differ from each other only at sequence positions 35–38 and, based on the structures of sbAvd-2 (I117Y) and sbAvd (I117Y) complexed with progesterone, position 35 seems to be the most important for binding.

Of these new antidins, sbAvd-7–9, enriched for binding progesterone from the AvLib-4 library, and hbAvd-4, enriched for binding hydrocortisone from the AvLib-5, were found to be the tightest progesterone binders analyzed so far. Based on a fluorometric analysis measuring intrinsic fluorescence, the affinities for progesterone were within the low nanomolar range: hbAvd-4, K_d_ = 39 nM; sbAvd-8, 11 nM; sbAvd-9, 13 nM; and sbAvd-7, 5 nM.

**Fig 5:**
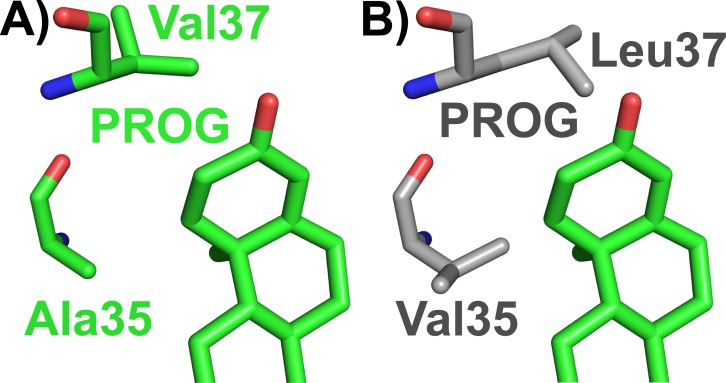
Suggested amino acid replacements in the ligand-binding site of the sbAvd-2 (I117Y)-progesterone complex structure (green carbon atoms; subunit I) [PDB:5LUR] likely to improve the progesterone-binding affinity of the sbAvd-2 (I117Y)-progesterone complex structure. (A) Ala35 and Val37 around the progesterone ligand (sticks) in the sbAvd-2 (I117Y)-progesterone complex structure being respectively replaced by (B) Val35 and Leu37 (sticks; grey carbon atoms). Nitrogen atoms are colored blue and oxygen atoms red.

Similarly to sbAvd-1–6 and sbAvd-2 (I117Y) [38], sbAvd-7–9 all have modified L1,2-loop residues 13–16 (RMNH) in comparison to chicken Avd (DLGS), supporting the idea of the importance of a histidine residue at position 16 for high-affinity progesterone binding and reduced biotin-binding affinity, in agreement with our sbAvd-2 (I117Y)–progesterone complex structure (see above). The differences between the previously characterized sbAvds and the new progesterone-binding antidins (sbAvd-7–9) are found at positions 33, 35–38 and 77. Based on our new X-ray structure of sbAvd-2 (I117Y), and amino acid sequence comparisons between sbAvd-2 and sbAvd-7 (Table 2), it seems likely that the RMNH-containing antidins benefit from a small aliphatic side chain, either of alanine or valine, at position 35 (sbAvd-2, 111 nM; sbAvd-5, 108 nM; sbAvd-6, 117 nM; sbAvd-7, 5 nM; Fig 5) and of an aliphatic residue, such as valine or leucine (sbAvd-2; sbAvd-4, 135 nM; sbAvd-9, 13 nM; sbAvd-7), or a residue with a small side chain, such as serine (sbAvd-6; sbAvd-8, 117 nM) at position 37. The residues at position 36 and 38 are not likely to directly affect progesterone binding. Moreover, an alanine residue at position 77 (only in sbAvd-7–9) may have a role in stabilizing the D-ring of progesterone and partially explain the higher affinity of the sbAvd-7–9 antidins for progesterone in comparison to other antidins. Furthermore, a phenylalanine residue (only in sbAvd-8 and sbAvd-10 out of the RMNH-containing antidins) instead of a tyrosine residue at position 33 may reduce the biotin-binding affinity [46], since a tyrosine residue at this position is highly conserved within all known Avds from different species. However, the effect of this position–tyrosine versus phenylalanine–on progesterone-binding affinity is not trivial to predict, and our docking analyses were unable answer this question (see below).

### Physico-chemical properties of sbAvd-7

**Fig 6:**
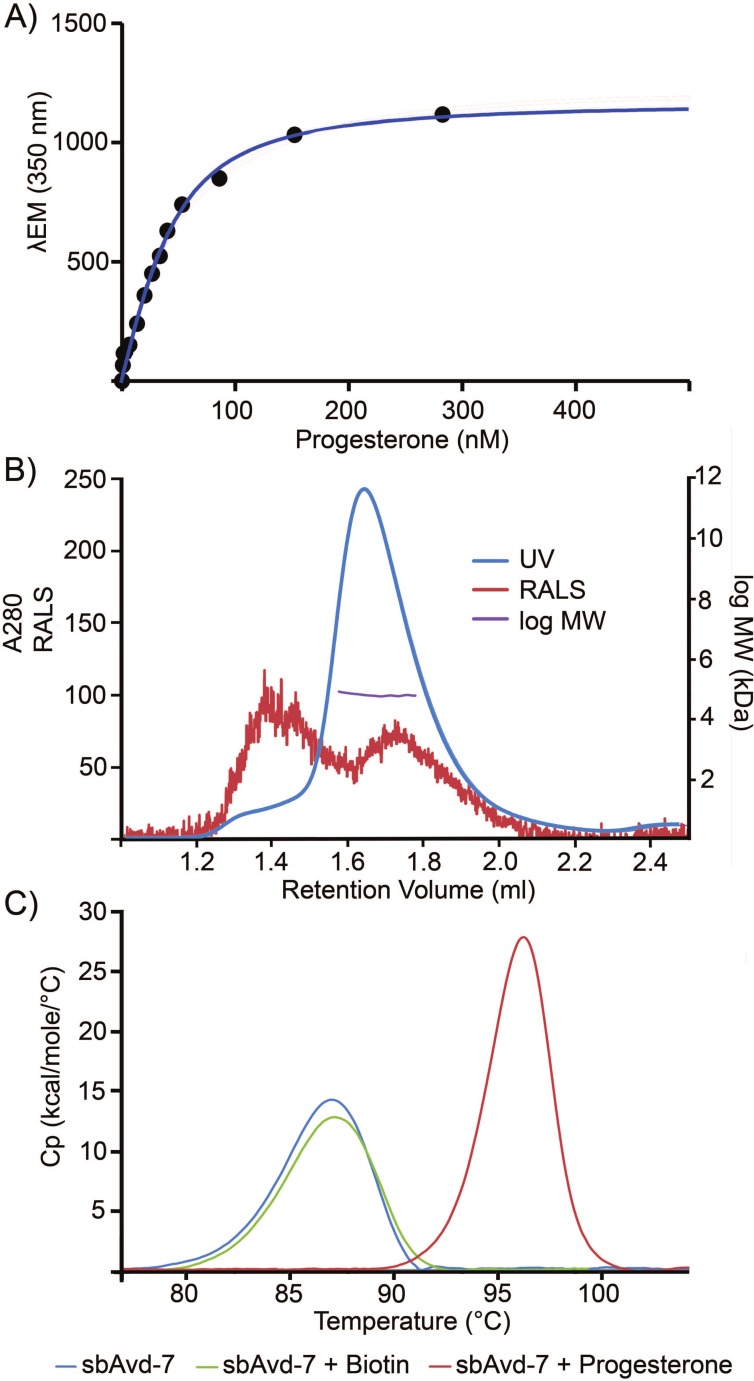
Biophysical characterization of sbAvd-7 for progesterone binding properties, oligomeric state and thermal stability. Binding affinity (K_d_ = 4.9 nM) was determined (A) measuring intrinsic fluorescence as a function of progesterone concentration. (B) LC-SLS analysis revealed a molecular weight of 65 kDa (Retention volume (RV) 1.647 ml) for sbAvd-7, which corresponds well to the molecular weight of the tetrameric protein calculated from the sequence (MW = 62.6 kDa). Retention volumes of the molecular weight standards: bovine serum albumin (66.7 kDa) 1.547 ml; carbonic anhydrase (29 kDa) 1.792 ml; alcohol dehydrogenase (150 kDa) 1.432 ml and beta amylase (200 kDa) 1.365 ml. (C) Differential scanning calorimetry. Progesterone increased the thermal stability of sbAvd-7, indicating tight interaction between sbAvd-7 and progesterone (ΔT_m_ = 10.23°C). In contrast, biotin showed no stabilizing effect (ΔT_m_ = 0.27°C).

The progesterone-binding properties of antidins were studied with a fluorometric assay, where quenching of the intrinsic fluorescence of the protein was measured as a function of the ligand concentration; a dissociation constant (K_d_) of 5 nM was determined for the progesterone-sbAvd-7 complex (Fig 6A).

The oligomerization state of sbAvd-7 was inspected with a combination of size exclusion chromatography (SEC) and UV-VIS/right angle light scattering (RALS). Based on SEC analysis, sbAvd-7 was found to be a tetrameric protein similarly to the wild-type chicken Avd. On SEC-UV-VIS/RALS analysis, sbAvd-7 was mainly tetrameric but also larger oligomeric forms were detected (Fig 6B); higher-order oligomeric forms are typically observed for tetrameric Avds in SEC (see for example [47]).

The T_m_ value of 85.84°C was measured for sbAvd-7 in the absence of ligand (Fig 6C) using differential scanning calorimetry (DSC). Progesterone clearly increased (ΔT_m_ = 10.23°C) the thermal stability of sbAvd-7 (Fig 6C), while biotin did not have an effect (ΔT_m_ = 0.27°C) on thermal stability (Fig 6C).

### Docking analysis of antidins

In order to better understand the atomic interactions that discriminate between stronger and weaker ligand-binding antidins, we conducted molecular docking analysis. We focused on the antidins sbAvd-7–9, which we found to be the highest affinity progesterone binders based on a fluorometry-based assay (see above).

**Fig 7:**
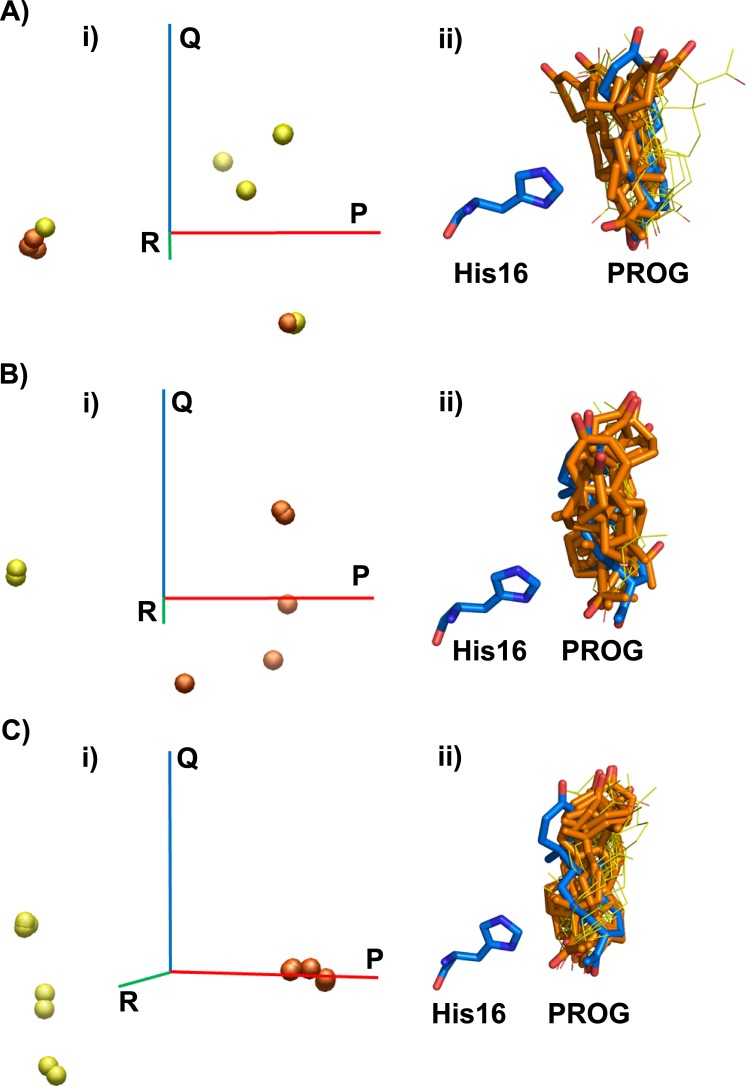
Principal component analysis (PCA) of RMSD values between all pairs of poses from the sbvd-7 (A), sbAvd-8 (B) and sbAvd-9–progesterone (C) docking experiment. i) The percentage of the total variance displayed on each PCA axis–P (red), Q (blue) and R (green)–is listed in S1 Table. The poses that were docked in a similar orientation as the progesterone ligand seen in the sbAvd-2(I117Y)–progesterone crystal structure–four for sbAvd-7, six for sbAvd-8 and seven for sbAvd-9 –are drawn as orange spheres and other poses as yellow spheres. ii) Comparison of the conformation and relative orientation of the progesterone poses from the docking experiment to the progesterone ligand of the crystal structure (thick sticks, blue carbon atoms). His16 of the crystal structure is also shown. The poses, which are in a similar conformation and orientation as the progesterone ligand in the crystal structure, are drawn as thick sticks (orange carbon atoms) and other poses as thin sticks (yellow carbon atoms). Nitrogen atoms are colored blue and oxygen atoms red.

Four out of eleven (sbAvd-7), six out of eight (sbAvd-8) and seven out of sixteen (sbAvd-9) progesterone poses (Fig 7, S1 Table) were found to be docked in a similar orientation, and as deep in the binding pocket, as the ligand was observed in the crystal structure of the sbAvd-2 (I117Y)-progesterone complex [PDB:5LUR]. However, despite extensive visual analysis of the ligand poses, we could not fully explain the difference in the binding affinities of sbAvd-2 (I117Y), sbAvd-7, sbAvd-8 and sbAvd-9 at the atomic level. The putative hydrophobic/van der Waals interactions, even for the docked progesterone ligands closely resembling the orientation of the progesterone ligand in the crystal structure of sbAvd-2 (I117Y), were not trivial to predict; the interaction networks varied among the different progesterone poses on each of the antidins sbAvd-7–9 despite the unique set of mutations in the L3,4 loop region, and at positions 33 and 77. For reference, we also docked the ligand from the crystal structure of the sbAvd-2 (I117Y)–progesterone complex [PDB:5LUR] back into the ligand-binding site and, even in this case, the docking produced varying poses dissimilar to the ligand observed in the crystal structure, indicating the challenge of docking progesterone into Avd-like binding pockets. Interestingly, Panek and coworkers [48] recently reported that progesterone docked into human mineralocorticoid receptor and bacterial monooxygenase in orientation (‘D-ring first’) different from that observed in the crystal structures of these proteins (“A-ring first”).

In addition to progesterone, biotin was also docked to the sbAvd-7–9 antidins. As expected based on the analysis of the sbAvd-2 (I117Y) crystal structures ([PDB:5LUR] and [PDB:4U46] [38]), His16 hinders the biotin binding to these antidins, and we predict it to do so in all antidins selected from the AvLib-4 library having the RMNH-sequence at their L1,2 loop. Moreover, the mutations in the L3,4 loop are likely to further reduce the biotin-binding affinity of sbAvd-7–9, especially in the case of sbAvd-9, in which the bulky tyrosine side chain at position 35 (threonine in chicken Avd) extends into the ligand-binding site.

### Detection of progesterone from serum samples

**Fig 8:**
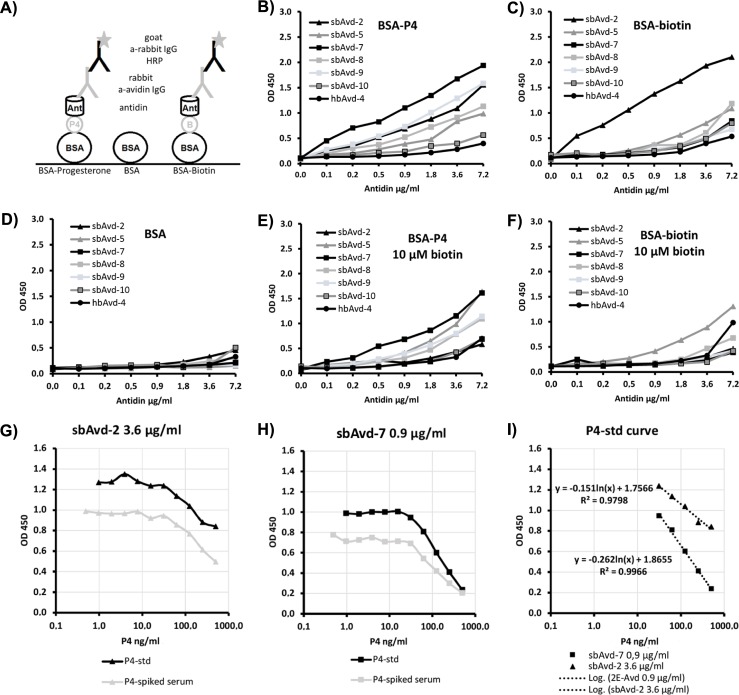
Determination of progesterone concentration using antidins. For the measurements, the antigen EIA measurement configuration (A) was used. The concentration dependency of the binding was then determined and, based on the results (B–F), antidins sbAvd-7 and sbAvd-2 were selected for the actual progesterone detection range measurements (G–I). The BSA conjugate of progesterone (P4) was: BSA-CMO-P4 (Sari Viitala, University of Eastern Finland).

The ability of antidins to detect progesterone was studied using a microplate-based assay (Fig 8A–8F). The effect of the antidin concentration for progesterone and biotin binding was studied using microplates coated with BSA-conjugated progesterone or biotin, and nonspecific binding using BSA-coated microplates. In order to study the residual biotin-binding affinity, free biotin was added to the samples to a final concentration of 10 μM (Fig 8A–8F). The binding of different concentrations of the antidins were measured using antibodies (primary antibody + HRP-conjugated secondary antibody). Based on this assay, the sbAvd-2 and sbAvd-7 antidins were found to be the best progesterone-binders and were thus selected for serum progesterone measurements. As shown in Fig 8G–8I, sbAvd-2 and sbAvd-7 can detect progesterone in phosphate buffer and in progesterone-spiked serum samples over a concentration range of 31.3–500 ng/ml.

## Discussion

Avd and streptavidin are widely applied in biotechnological applications because they enable the attachment of biotinylated molecules on a wide array of materials. The structure of the ligand-binding site of (strept)avidin has characteristics optimal for the recognition of small ligands: the binding site is deep and narrow in structure, and the loops at the entrance of the binding-site isolate the bound ligand from the surrounding environment. The aim of this study was to utilize and engineer the biotin-binding site of Avd to develop artificial Avd-based receptors, antidins, for small molecules other than biotin, and in particular for steroids. Furthermore, we aimed to significantly improve the affinity of the previously developed antidins towards progesterone. For this purpose, a synthetic DNA library with tailored randomization was created using TRIM technology. The DNA library was subcloned into phage display vector using Gateway technology and resulted adequate sequence coverage as compared to theoretical library size.

**Fig 9:**
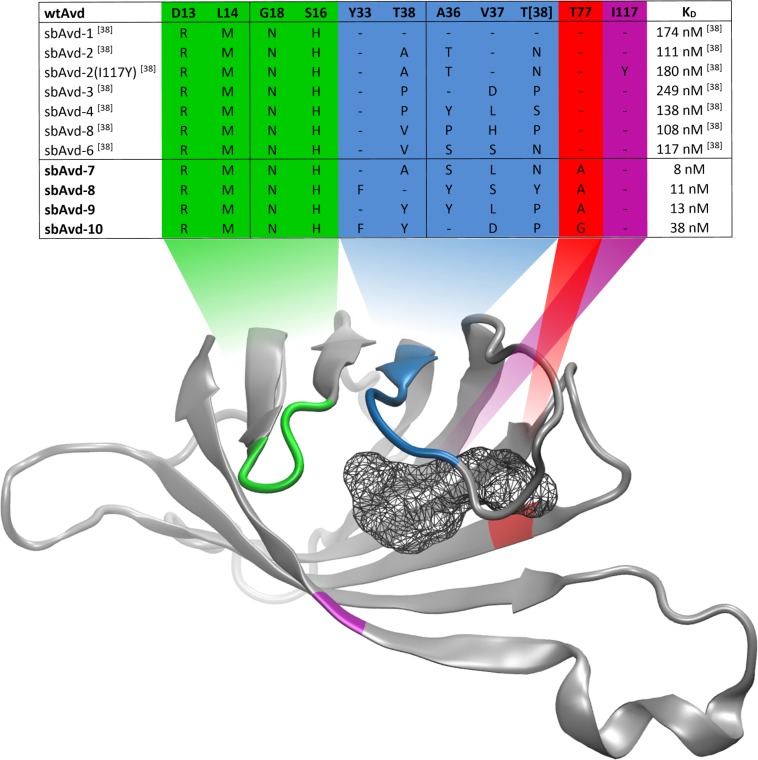
Summary of mutations introduced to the antidins sbAvd-1–10 and K_d_ values for their progesterone binding. The mutated regions/sites are indicated by different colours in the X-ray structure of wt avidin [PDB 2AVI; [19]]; only a monomer is shown for simplicity. The K_d_ values for progesterone binding were determined using intrinsic fluorescence quenching method.

Rational engineering of novel, artificial receptor-ligand pairs is highly dependent on detailed, atomic resolution information about protein-ligand interactions. Here, we solved the structure of the sbAvd-2 (I117Y) antidin [PDB:5LUR] co-crystallized with progesterone and carried out physicochemical analyses in order to help further improve the binding affinity of progesterone to sbAvd-type antidins (Fig 9). The solved X-ray structure pinpoints the importance of Met14 and His16 in the L1,2 loop for high-affinity progesterone binding and low-affinity biotin-binding, and the open conformation of the L3,4 loop hints for optimization of the amino acids of this loop for even higher affinity for progesterone. SbAvd-7, the highest affinity (5 nM) progesterone binder, has an alanine residue at position 35 and a leucine residue at position 37 suggesting that hydrophobic residues are needed at these positions for higher levels of progesterone affinity. Our docking analyses, even though not trivial, are also in agreement with the predictions based on the crystal structure of sbAvd-2 (I117), *i*.*e*. supporting the idea that the residues at position 35 and 37 of the L3,4 loop in particular, as well as at position 14 and 16 of the L1,2 loop, are critical for ligand specificity and affinity. However, it remains to be seen whether the L3,4 loop of sbAvd-7 containing residues Ala35 and Leu37 is in the open or closed conformation, or if it is possible to further optimize the residues at position 35 and 37 for tighter progesterone binding. Optimization of residues of the β6-strand (*e*.*g*. at position 77) or from the L5,6 loop (*e*.*g*. at position 73 and 75) may also improve the progesterone binding of the sbAvds; for example, mutating Ala77 of sbAvd-7–9 to a valine residue (threonine in sbAvd-2 (I117Y)) or mutating one of the serine residues at position 73 and 75 to a small hydrophobic residue while leaving the other serine for H-bonding with the 3-keto group of progesterone. Mutational analysis of residues especially at positions 14, 16, 35, 37, 73, 75 and 77, and crystallization trials of the sbAvd-7–9-progesterone complexes, would help address these questions.

Our physicochemical analysis of sbAvd-7 (Fig 6)–the tightest progesterone binding antidin produced to date–showed that the engineered avidin is tetrameric similarly to the wild-type protein. Binding of progesterone stabilized sbAvd-7, in agreement with the measured K_d_ of 5 nM. In contrast to wt Avd, which is strongly stabilized by biotin [39], biotin had a negligible effect on the thermal stability of sbAvd-7, which reflects a significant loss in biotin-binding affinity. Altogether, our findings suggest that avidin tolerates genetic engineering and can be manipulated in terms of ligand-binding specificity, properties that should enable the future development of binders for various small molecules.

We demonstrated that sbAvd-2 and sbAvd-7 can detect progesterone from serum samples down to a concentration of ~30 ng/ml and hence has potential to be used in diagnostics. However, to test *e*.*g*. breeding times in dog, higher sensitivity and a wider detection range is needed–the progesterone levels in domestic female dog serum ranges between 0.1–60.0 ng/ml, and the most important measurement range is between 1.0–10.0 ng/ml [49].

The vast amount of reagents already available for developing the (strept)avidin-biotin-system, including antibodies, chromatography resins etc. and constant need for improved performance should help drive development of novel Avd-based technologies, including the antidin-steroid protein-ligand pair as a molecular tool. In comparison to the classical (strept)avidin-biotin-system, the novel antidins described here are not affected *e*.*g*. by endogenous biotin (Fig 8) and, in comparison to antibodies, the high stability of antidins is a clear advantage and may enable the use of antidins in applications where antibodies cannot be used because of stability issues. Furthermore, the expression of antidins in *E*. *coli* is cost-efficient and straightforward, and therefore one may envision applications for antidins in methods such as chemical synthesis and sample pre-processing.

## Materials and methods

### Homology modeling

Two different homology models were built for each of the sbAvd-7–9. Firstly, models to study progesterone binding were built using the sbAvd-2 (I117Y)–progesterone complex structure [PDB:5LUR] as a template. The L3,4 loop of this crystal structure is in the open conformation and the residues Val37-Asn42 (subunit I and III) or Val37-Glu43 (subunit II and IV) were missing from the structure and therefore built using the Crosslink proteins panel from the Biologics suite (Maestro 11.3, release 2017–3, Schrödinger, LLC, New York, NY, 2017). The conformation of the L3,4 loops were predicted by searching through the curated structures in the PDB database [50,51] and energy calculations were made using PRIME (Maestro 11.3). Secondly, models to study biotin binding were done using two templates: chicken Avd–biotin complex structure [PDB: 1AVD] [52] with the L3,4 loop in the closed conformation was used as the template structure for all residues except for residues 13–16 of the L1,2 loop for which the sbAvd-2 (I117Y)-progesterone complex structure [PDB:5LUR] was used as the template. Structure-based sequence alignment was made using the program Malign [53] in Bodil [54]. The homology models were created using Modeller 9.14 [55]. Ten tetrameric models were created for each antidin and the models were ranked using the Molecular PDF (molpdf) score and Discreet Optimized Protein Energy (DOPE) score automatically produced by the program. MolProbity [56] was used for checking the stereochemical and geometrical parameters of the final models. More than 96% of the residues of all the models created were in the favoured regions in Ramachandran plots [57]. Final model selection for docking was done based on visual analysis in PyMOL (The PyMOL Molecular Graphics System, Version 1.8 Schrӧdinger, LLC) and the Ramachandran plots [57].

### Molecular docking

Biotin (Pubchem Compound ID: 171548) and progesterone (Pubchem Compound ID: 5994) were retrieved from the NCBI PubChem database (https://pubchem.ncbi.nlm.nih.gov/) and were imported into the Maestro 11.3 (Release 2017–3: Schrödinger, LLC, New York, NY, 2017) for minimization using the OPLS_2005 force field in the LigPrep wizard. Possible ionization states were generated at the target pH of 7.0 ±2.0 using Epik. Counter ions and/or water molecules were removed from the ligand structure and possible tautomers were generated. Geometrical irregularities in the ligands were corrected using the option ‘Retain specified chiralities’.

The Protein Preparation wizard in Maestro 11.3 was used to prepare the progesterone-removed crystal structure of sbAvd-2 (I117Y) [PDB:5LUR] with modeled L3,4 loop (only for progesterone docking) and the selected sbAvd-7–9 models (see above) for progesterone and biotin docking; All protein structures were optimized and minimized using the force field OPLS_2005. Water molecules beyond 5 Å from any amino acid residues were deleted from the heteroatom groups and H-bond assignments were done at the default pH of 7.0 using PROPKA. The amino acid residues at the ligand-binding site equivalent to 16, 39 and 73 of chicken Avd [PDB: 1AVD] were chosen as the centroids of the receptor (sbAvd-2 (I117Y), sbAvd-7–9) for ligand binding with automatic box size determination by the Induced Fit Docking (IFD) wizard [58–60], which utilizes both GLIDE and PRIME programs. The standard protocol was applied and ring conformations were sampled in an energy window of 2.5 kcal/mol by selecting the ‘Sample ring conformation’ option, which samples the rotatable bonds, while leaving the backbone fixed. Amide bonds were allowed to be in cis or trans conformation. In the GLIDE docking tab, the receptor and ligand van der Waals scaling were both kept at the default value of 0.5 to permit enough flexibility for the amino acid side chains and the ligand to dock in the best possible poses. A maximum of 20 poses were allowed per ligand. Amino acid residues having atoms within 5.0 Å of the ligand poses along with their side chains were refined and optimized using the PRIME refinement option. In the redocking option of GLIDE, the extra precision (XP) option was selected and redocking of ligands in a structure was allowed within 30 kcal/mol of the best docked conformation, for a maximum of 20 structures. The results were categorized based on parameters such as the docking score, GLIDE Gscore, PRIME energy and IFD score.

### Principal component analysis

Principal component analysis (PCA) was used to display the relationships among the docked poses of two ligands–biotin and progesterone–within the binding site of the antidins. For each ligand, the root mean-squared distance was computed between all pairs of poses over all ligand atoms using a C program fixrmsd (Johnson, MS; unpublished). For each ligand, the square matrix of distances calculated among the poses was input to the program PCA (Johnson, MS; unpublished) and coordinates representing each pose in three dimensions were output as a PDB formatted files for visualization using the program Bodil [54].

### Crystallization and data collection

The sbAvd-2 (I117Y) mutant (1.8 mg/ml; 20 mM sodium phosphate, 1 M sodium chloride, 20 mM imidazole, pH 7.4) was co-crystallized with 50 mM progesterone in 10:1 (v/v) ratio using the vapour diffusion method. Sitting drops (150 nl of the protein-ligand solution + 75 nl of well solution) were prepared with the mosquito liquid handling robot (TTP Labtech). The well solution (0.18 M sodium chloride, 0.09 M sodium cacodylate (pH 6.5) and 1.8 M ammonium sulfate) used was derived from the commercial JCSG-plus crystallization screen (NeXtal Tubes Suites, Qiagen, USA). The crystal used for data collection appeared within a few weeks of incubation on 96-well triple sitting drop iQ plates (TTP Labtech) at 21°C in a temperature controlled crystallization incubator (RUMED model 3201). The X-ray diffraction properties of the crystals were initially analysed using the PX Scanner (Agilent Technologies). For data collection, 1 μL of cryoprotectant (12.5% di-ethylene glycol, 12.5% MPD, 37.5% 1,2-propanediol, 12.5% DMSO; CryoProtX, Molecular Dimensions) was added to the crystallization drop just prior to freezing in liquid nitrogen. Data were collected at ESRF beam line ID30A-3, Grenoble (Table 1). Data were processed using XDS [40].

### Structure determination and refinement

Initial phase estimates for the structure factors were obtained using the molecular replacement program Phaser [61] within the CCP4i GUI [62–64]. The Matthews coefficient [65] predicted the presence of two molecules per asymmetric unit. For molecular replacement, a monomer of the apo sbAvd-2 (117Y) structure [PDB:4U46] [38] was used as the template. The space group of the structure was confirmed to be *P*2_1_2_1_2 after the replacement. The X-ray structure was refined with Refmac5 [41] and edited/rebuilt using Coot [66]. Solvent atoms were added to the structure and the final validation was done using the inbuilt tools of Coot [66] and MolProbity [56]. PyMOL and Bodil [54] were also used to check the final structure, and for creating figures. The coordinate file and structure factors were deposited in the Protein Data Bank [50,51] with the PDB code 5LUR.

### Phage display libraries

Two new synthetic Avd gene libraries (AvLib-4–5 libraries, see Table 2) were designed based on the enriched sequences from the previous study [38] and synthesized by Thermo Fisher Scientific Inc. The library AvLib-4 was designed for progesterone panning and is based on the enriched sbAvd-2–6 sequences from AvLib-3 [38], whereas the library AvLib-5 was designed for hydrocortisone panning and is based on the enriched hbAvd-1–2 and cabAvd-1–2 sequences from AvLib-2 [38]. However, we applied limited diversity (in the most extreme case binary sequences of Tyr/Ser) to our library design, inspired by [67,68] in which this technique was successfully used to generate high-affinity and specific protein-protein interactions. The synthetic gene library was constructed using either the chicken Avd gene or its mutant, called sbAvd-1: the genes were diversified using preassembled trinucleotide building blocks within the chemical synthesis process (the TRIM (trinucleotide mutagenesis) technology) enabling the introduction of a restricted set of amino acid residues in the L3,4 loop or in the loops L1,2 and L5,6 (Table 2). Additionally, three amino acid residues essential for biotin-binding [19], were randomized allowing for all of the amino acid residues except cysteine (Table 2). The total number of mutated amino acid residues was eleven or nine. The Shine-Dalgarno (AGAAGGAGATATACAT) and OmpA signal sequences [47] preceded the avidin gene, and the library was flanked with *att*L sequences in order to enable Gateway (LR) cloning into a compatible phagemid vector [42]. For optimal protein expression, the sequence was codon-optimized for *E*. *coli*. The synthetic libraries were delivered as an amplified and non-amplified library. The quality of the library had been analyzed by the manufacturer (Thermo Fisher Scientific Inc.) using bulk sequencing.

The phagemid libraries were constructed by LR-cloning the amplified library with the pGWSacBRphagemid vector (1:1 molar ration) in overnight reactions, and subsequently transformed into *E*. *coli* XL1-Blue cells by electroporation in parallel reactions (essentially as described earlier in [42]). A sample of each reaction was plated enabling transformation efficiency calculations after which the parallel reactions were pooled together and diluted 1:10 with SB medium supplemented with ampicillin (100 μg/ml), tetracycline (10 μg/ml), sucrose (10% (w/v)) and glucose (2%). The bacterial culture was allowed to grow in a shaker for 1 h, after which glycerol stocks were prepared. The rest of the culture was used for plasmid extraction, as described in [42]. Briefly, the biopanning was performed using the automated magnetic bead platform, Precipitor (Abnova, Taipei City, Taiwan) essentially as described in [42] using BSA-conjugated ligands, cortisol-3 BSA conjugate (C037, CalBioreagents Inc.) or progesterone-3-CMO:BSA (prepared by S. Lehtonen and confirmed to contain approximately 3 progesterone molecules per BSA) as target molecules.

### Selection of the antidins

Three rounds of selection were performed for the AvLib-4 library specifically designed for progesterone binding. Four different phage production conditions were tried in parallel (1: in the presence of 0.1% glucose to repress the Avd production; 2: 0.1% glucose + biotin (2 g/L) to reduce the toxicity effect of Avd production; 3: 0.1% glucose + 10 mM IPTG to induce protein production; 4: 0.1% glucose + biotin (2 g/L) + 10 mM IPTG), while keeping the elution conditions constant. For biopanning, a magnetic bead platform, Precipitor (Abnova), was used with carboxyl magnetic beads (Abnova) coated according to instructions from the manufacturer. The elution strategy was inspired from the [45], using an excess of free progesterone (20 μM) in the first round elution and continuing the next two panning rounds by using nonspecific acidic elution [38] instead.

First, the coated beads were blocked with 2.5% milk-PBS (procedure described in [38]), and additionally also with 2% w/v BSA in PBS/T for 1 h (according to [45]) instead of the depletion step with BSA (as described in [38]). The library AvLib-4 *E*. *coli* XL1-Blue cell culture was grown from glycerol stock essentially as described in [69] in the presence of 100 μg/ml ampicillin, 10 μg/ml tetracyclin, 10% sucrose (w/v) and 2% glucose (w/v). The following day, the cell culture was diluted 1/50 and grown in a 10 ml-culture volume in SB+amp+tet+gluc (1%) in four Erlenmeyer bottles (enabling four different culture conditions in parallel) until the OD_600_ = 0.7–1. Helper phage coinfection was followed by dilution of the 10 ml-infections to 100 ml-culture volume and grown as described previously. At this point the glucose concentration was lowered to 0.1%. After 30 minutes, for two of the cultures, biotin (2 g/L) was added. After 2 h-incubation, kanamycin (70 μg/ml) was added, the temperature was reduced and the cultures were grown +28°C O/N. Additionally, for two of the cultures, IPTG (10 mM) was also added. Thus, four different culture conditions were used in parallel. The following day, the phages were collected by PEG-precipitation essentially as described in [69]. The first round elution was performed with 20 μM progesterone in PBS for 30 min followed by phage production conditions described above and PEG precipitation (essentially as described in [69]), whereas subsequent elution rounds and phage productions were performed as described in [38].

For hydrocortisone selection, two sets consisting of three rounds of panning were performed with the AvLib-5 library using cortisol-3-BSA (CalBioreagents). At first, the used panning conditions were inspired by those described in [70], but here all three different elution conditions (free ligand, acidic and basic elution) were performed in each round and always pooled together. First, both conjugated magnetic beads and phages were blocked with 2% BSA in PBS-Tween (0.05%) overnight +4°C and the phages were subsequently subjected to biotin preincubation for 30 min (RT, 250 rpm) using biotin-BSA (Leppiniemi, J., University of Tampere; 270 ng/well) -coated Nunc MaxiSorp 96-well plate (eBioscience Inc., San Diego, CA, USA). The bead-bound phages were eluted with moderate mixing speed with free hydrocortisone (100 μM) in PBS-Tween (0.05%) for 30 minutes, after that with 0.1 M trimethylamine base for 6 min and lastly with 0.1 M glycine/HCL pH 2.2 for 8 min. The base and acid elutions were subsequently neutralized with 4.75 μl of 2 M acetic acid and 14.3 μl of 0.5 M Tris base, respectively, and the eluates were pooled together and used for infecting 900 μl exponentially growing *E*. *coli* XL1-Blue cells. The phages were thus amplified on microplates essentially as described in [71]. The subsequent rounds were performed directly from the phage culture supernatants (essentially as described in [38]) similarly without biotin-preincubation step. This panning condition yielded the enrichment of antidins hbAvd-4 and hbAvd-5.

Later, hydrocortisone-panning was repeated for the pooled library consisting of both the AvLib-4 and AvLib-5 libraries and, again, three rounds of selection was performed. For elution, however, two different conditions were used: trypsin (1 mg/ml in PBS, 30 min) or free hydrocortisone (100 μM in PBS, 1 h). This time the phage amplifications after each panning round were carried out in Erlenmeyer flasks followed by PEG precipitation (essentially as described in [69]). Several clones were picked and tested using ELISA for their binding preference for cortisol-3 BSA against BSA and BSA-biotin. Further, the clones with strong ligand binding were selected based on biolayer interferometry (BLI; see supplementary data for the details) and fluorometry. All of the positive clones from the primary selection were sequenced, which resulted in antidins hbAvd-6–9.

### Protein production and purification

The most enriched Avd mutants from the hydrocortisone and progesterone pannings were subcloned with the OmpA signal peptide [47] and C-terminal 6xHis-tag into the pET101/D expression vector (Invitrogen) [37]. Proteins were expressed in *E*. *coli* strain BL21-AI (Invitrogen) or OverExpress C43(DE3) (Lucigen) essentially as previously described [47]. The fresh transformants were cultured in Lysogeny broth (LB) medium with 0.1% (w/v) glucose and ampicillin (100 μg/ml) at 28°C on a shaker until the culture reached OD_600_ 0.4. Protein expression was then induced by adding 0.2% (w/v) L-arabinose and 1 mM IPTG and the cultivation was continued for an additional 18 hours. Finally, the cells were collected by centrifugation (5000 g, 10 min, 4°C) and frozen.

The proteins were purified using affinity chromatography on a Ni-NTA (QIAGEN) column [37]. The bacterial cell pellets were suspended in binding buffer (20 mM NaH_2_PO_4_/Na_2_HPO_4_, 1 M NaCl, 20 mM imidazole, pH 7.4) and lysed by homogenization (EmulsiFlex-C3 homogenizator, Avestin Inc.). After pooling the elution fractions together, the imidazole concentration was reduced back to 20 mM using step-wise dialysis, and subsequently into 50 mM NaH_2_PO_4_/Na_2_HPO_4_, 650 mM NaCl, pH 7. The purity and quality of the isolated proteins was analyzed by UV/Vis spectrophotometry, SDS-PAGE, Western blotting with polyclonal anti-avidin antibody (University of Oulu, Finland) and dynamic light scattering–these analyses indicated that the quality of the purified proteins were comparable to that of the bacterially expressed Avd [47], which was used as a reference protein in the assays.

### Determination of progesterone concentration from serum samples

Dog serum samples were collected in a study to monitor hormone metabolites in dogs. The study plan CW 14/122 was approved by the ClinVet Committee for Animal Ethics and Welfare (CCAEW). The antigen EIA method was performed essentially as in [38]. At first, Avd- and progesterone-specific polyclonal antibody were tested in parallel, but since the Avd-specific antibody worked better with the antidins, it was selected for further use. Microwell (NUNC Maxisorp) plates were coated with BSA (500 ng/well; bovine serum albumin, A3059 Sigma-Aldrich), progesterone-BSA conjugate (500 ng/well, BSA-CMO-P4, Sari Viitala, University of Eastern Finland) and BSA-biotin (500 ng/well; Jenni Leppiniemi, University of Tampere) in 10 μM NaHCO_3_ (pH 9.5) at +37°C for 2 h. After washing the wells (three times with PBST), the plates were blocked with 0.5% BSA in PBS at +37°C for 30 min. After washing the wells once, the serially diluted antidins (0–7.2 μg/ml) in 0.5% BSA containing sodium phosphate buffer (50 mM NaH_2_PO_4_, 650 mM NaCl, pH 7.0 for hbAvd-4, sbAvd-7–10; and 20 mM NaH_2_PO_4_, 1 M NaCl, pH 7.4 for sbAvd-5 and sbAvd-2) were added in a volume of 100 μl/well with and without inhibitor (10 mM D-biotin, 0340 VWR), and incubated at +37°C for 1 h. Followed by three PBST washes, polyclonal anti-avidin IgG (University of Oulu; 1:5000 dilution) was added (100 μl/well) and the wells were incubated at +37°C for 1 h. After washes, the secondary antibody (HRP-conjugated anti-rabbit IgG 1:10 0000, 611–1322 Rockland) was added 100μl/well and incubated at +37°C for 30 min. After the final washing step, 100 μl/well of 3,3´, 5,5´ tetramethylbenzidine substrate solution was added and the color was allowed to develop at room temperature for 30 min. The reaction was stopped by adding 50 μl of 2 M H_2_SO4 and the optical density was determined at 450 nm using a microplate reader (*ELx800* BioTek Instruments, Inc, USA).

The sbAvd-2 and sbAvd-7 antidins were used for the progesterone binding assay. Wells were coated with BSA-CMO-P4 and blocked as described above. Progesterone was serially diluted (0–1 μg/ml) in sample buffer (0.5% BSA, 50 mM NaH_2_PO_4_, 650 mM NaCl, pH 7.0) and in pre-diluted serum sample (1:25 in sample buffer). SbAvd-2 and sbAvd-7 were diluted in sample buffer for concentrations of 3.6 μg/ml and 0.9 μg/ml, respectively, and 100 μl/well of the progesterone dilutions were added. Wells were incubated at +37°C for 1 h. The binding was detected by the method described above.

### Progesterone-binding analysis of selected antidins with fluorometric assay

The binding affinity of the unconjugated small molecules to antidins was determined utilizing the intrinsic fluorescence originating from the aromatic amino acid residues (mainly tryptophan and tyrosine) of Avd and the fluorescence quenching caused by ligand binding. In brief, 100 nM protein samples in 50 mM NaH_2_PO_4_/Na_2_HPO_4_, 650 mM NaCl, pH 7 were excited at 280 nm, and emission was collected at 350 nm using QuantaMaster Spectrofluorometer (Photon Technology International, Inc.) with 2 nm slits. The assay was performed in a quartz cuvette with stirring at 25°C. The ligand was added to the protein sample in small aliquots (6–50 000 nM) and the fluorescence intensity was monitored after a short incubation. The dissociation constant (K_d_) was determined from the resulting quenching curve using GraphPad Prism (GraphPad Software, Inc.). The data were fitted to a quadratic equation [72] for tight binding interactions, which takes ligand depletion and nonspecific binding into account (described in detail in [38]) (Fig 6).

### Differential scanning calorimetry (DSC)

The thermal stability of the antidin sbAvd-7 in the presence and absence of ligands was analyzed using an automated VP-Capillary DSC System (Malvern, Microcal Inc.) essentially as described in [73]. Protein samples in the sodium phosphate buffer (50 mM, pH 7) containing 650 mM NaCl were degassed prior to the measurement. The protein concentration in the cell was 6.4 μM, and the molar concentration of the ligands (D-biotin (Biochemica, Fluka) and progesterone (Steraloids Inc., USA)) were three times higher (Fig 6).

### Size exclusion chromatography with static light scattering (SEC-SLS)

The oligomeric state of the antidin sbAvd-7 was analyzed with size exclusion chromatography (SEC) using a liquid chromatography instrument (CBM-20A, Shimadzu Corporation) equipped with an autosampler (SIL-20A), UV-Vis (SDP-20A), and a fluorescence detector (RF-20Axs). The instrument was integrated with a static light scattering instrument (SLS, Zetasizer μV light scattering detector (Malvern Instruments Ltd.)) to determine molecular weight of the eluted proteins. The instrument was controlled using Lab Solutions Version 5.51 (Shimadzu Corporation) and OmniSEC 4.7 (Malvern Instruments Ltd.). Samples (~50 μg in 10–100 μl) were injected onto a Superdex200 Increase 5/150GL column (GE Healthcare) and equilibrated with the buffer the protein was dialyzed against (50 mM sodium phosphate, 650 mM NaCl, pH 7) with a flow rate of 0.1 ml/min at 20°C. Molecular weight determination was done by calculating a standard curve based on the elution volume of the molecular weight markers (CA, carbonic anhydrase 29 kDa; BSA, Bovine Serum Albumin 66 kDa; ADH, Alcohol Dehydrogenase 150 kDa; BA, ß-Amylase 200 kDa, Sigma-Aldrich), and alternatively, using the light-scattering intensity-based determination protocol involving BSA (monomeric peak) in SLS detector calibration using a Malvern microV detector and the OmniSEC software (Malvern Instruments Ltd.) (Fig 6).

## Conclusion

We have used directed evolution and rational mutagenesis to develop new antidins with high affinity towards progesterone. These antidins have high stability and could, in the future, expand the limited pool of useful scaffolds capable of binding small molecules with high specificity and affinity and even challenge traditional antibodies in *e*.*g*. diagnostic applications. In this regard, we have demonstrated that the highest affinity (K_d_ ~ 5 nM) progesterone-binding antidin, sbAvd-7, can be used to measure progesterone in serum samples. In order to better understand the progesterone-binding mode of the antidins, and as an aid to further optimize the binding and physicochemical properties of antidins, we characterized the crystal structure of the sbAvd-2 (I117Y)–progesterone complex [PDB:5LUR].

## Supporting information

S1 Fig Vector map of the constructed pGWSacBRphagemid and the schematic representing the Gateway LR cloning of the new synthetic DNA libraries into the phagemid vector. (TIF)

S1 Table Percent of total variance displayed over the first three dimensions in the PCA analysis of the pairwise RMSD values among poses for the docked ligands. (DOCX)
