# Supplementary figures and images for: Molecular features of steroid-binding antidins and their use for assaying serum progesterone
Source: PLoS One. 2019 Feb 20;14(2):e0212339. doi: 10.1371/journal.pone.0212339 (PMC6382169; doi:10.1371/journal.pone.0212339)

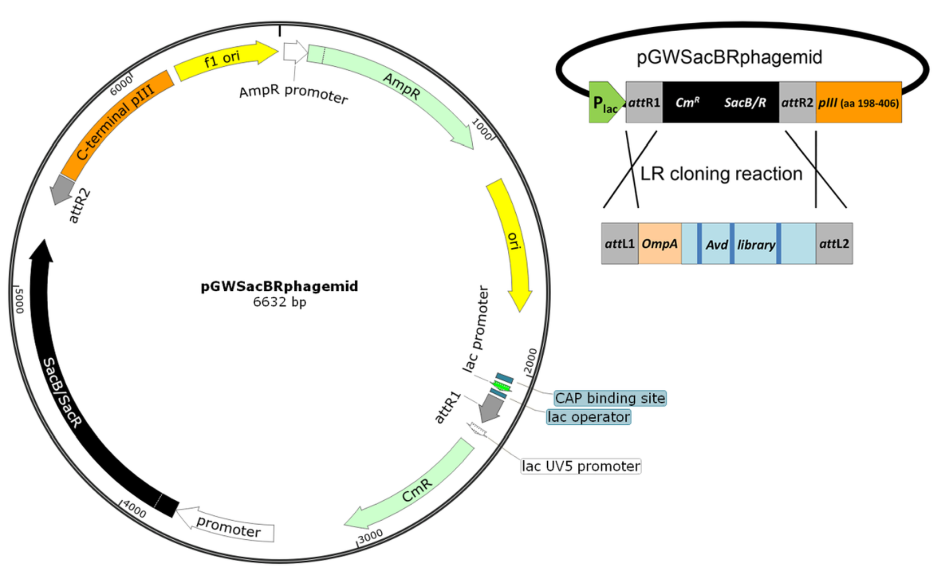

Supplement: S1 Fig — (TIF) [file pone.0212339.s001.tif]
