# Supplementary material for: Molecular features of steroid-binding antidins and their use for assaying serum progesterone
Source: PLoS One. 2019 Feb 20;14(2):e0212339. doi: 10.1371/journal.pone.0212339 (PMC6382169; doi:10.1371/journal.pone.0212339)
Supplement: S1 Table — (DOCX) [file pone.0212339.s002.docx]

**Supporting information**

Functional screening of the avidin variants

In order to rank the new avidin variants (antidins), clones from the 3^rd^ and 2^nd^ panning rounds were tested for their progesterone and hydrocortisone binding, and for biotin and BSA binding (negative control). The clones with increased progesterone or hydrocortisone affinity, and decreased biotin and BSA binding affinity, were sequenced and selected for further ligand-binding analyses using protein microplate assays and BLI biosensor measurements.

Protein microplate assays

The ligand-binding specificity of selected antidins was determined with a microplate assay essentially as described in [1]. In the assays, the wells were coated with 125–500 ng of BSA-conjugated ligands (Cortisol-3 BSA conjugate, C037, CalBioreagents Inc.; Progesterone-3-CMO:BSA, Q2606-000, Steraloids Inc.; or biotinylated BSA (Jenni Leppiniemi, University of Tampere)). BSA was used as a negative control. Antidins were expressed in *E. coli* and either the periplasmic fraction of the cell pellet (prepared simply by centrifuging a twice freeze-thawed cell pellet resuspended in PBS buffer) or purified protein (with concentration of 0.2–200 nM) were used as samples. For detection, either the alkaline phosphatase (AP) assay or the horse radish peroxidase (HRP) assay were used.

Biolayer interferometry (BLI) biosensor measurements

The enriched antidins were analyzed from periplasmic fractions of cell pellets also using BLI biosensors on Octet384 (Pall ForteBio). Similarly to the microplate assay, this method requires conjugated small molecule ligands. In addition to the relative binding level at equilibrium, BLI biosensor method allows the analysis of the binding kinetics, too. The AR2G (carboxylate functionalized amine reactive 2^nd^ generation) sensor surfaces were covalently coupled with BSA or BSA-conjugated ligands (Cortisol-3 BSA conjugate, C037, CalBioreagents Inc.; Progesterone-3-CMO:BSA, Q2606-000, Steraloids Inc.) at a concentration of 20 μg/ml (in acetate buffer pH 3.5) via EDC/s-NHS (0.1 M/0.05 M) mediated amide bond formation. PBS buffer with Tween-20 (0.01%) and BSA (1 mg/ml) was used as a liquid phase in all of the following BLI sensor functionalization steps. The temperature was fixed to 25°C and the stirring speed was set to 500 rpm. Black, tilted-bottom 384-well plates (Pall ForteBio, Menlo Park, USA) were used for sample measurement to ensure minimal drift in the measured signal between the analysis steps. Typically, a BLI running set-up with covalently coupled AR2G sensors and antidins was performed as follows: 1) The baseline of the sensors and device in dH_2_O without any binding events was recorded for 1 min. 2) EDC/s-NHS activation in dH_2_O for 10 min. 3) Washing step with dH_2_O to remove excess EDC/s-NHS for 1 min. 4) Protein was immobilized at a concentration of 20 μg/ml in acetate buffer pH 3.5 for 25 min. 5) Excess of sulfo-NHS esters were quenched using 1 M ethanolamine for 5 min. 6) The sensors were incubated in the buffer for 5 min to measure the baseline of the sensors. 7) The antidin association from periplasmic fractions were measured to the BSA-conjugated ligands covalently coupled to the AR2G sensors for 10 min, followed by 8) dissociation step of 10 min in measurement buffer alone or with free ligand. 9) Washing step with measurement buffer for 5 min.

**S1 Table: Percent of total variance displayed over the first three dimensions in the PCA analysis of the pairwise RMSD values among poses for the docked ligands**

| Protein | Ligand | Variance along P-axis (%) | Variance along Q-axis (%) | Variance along R-axis (%) | Total Variance represented in the 3 axes (%) |
| --- | --- | --- | --- | --- | --- |
| sbAvd-7 | Progesterone | 59.69 | 19.25 | 11.3 | **90.24** |
|  | Biotin | 35.29 | 28.8 | 12.33 | **76.42** |
| sbAvd-8 | Progesterone | 51.9 | 23.83 | 16.22 | **91.95** |
|  | Biotin | 49.12 | 20.22 | 10.46 | **79.8** |
| sbAvd-9 | Progesterone | 81.78 | 8.15 | 4.54 | **94.47** |
|  | Biotin | 38.27 | 26.87 | 10.53 | **75.67** |

1. Riihimäki TA, Hiltunen S, Rangl M, Nordlund HR, Määttä JAE, Ebner A, et al. Modification of the loops in the ligand-binding site turns avidin into a steroid-binding protein. BMC Biotechnol. 2011;11:64.
